# 3D density imaging using gravity and gravity gradient in the wavenumber domain and its application in the Decorah

**DOI:** 10.1038/s41598-023-49711-z

**Published:** 2024-01-02

**Authors:** Huiyou He, Jian Fang, Dongmei Guo, Ronghua Cui, Zhixin Xue

**Affiliations:** 1 State Key Laboratory of Geodesy and Earth’s Dynamics, Innovation Academy for Precision Measurement Science and Technology, Chinese Academy of Sciences, 340 Xudong Road, Wuhan, 430077 China; 2 Wuhan Gravitation and Solid Earth Tides National Observation and Research Station, Wuhan, 430071 China; 3 University of Chinese Academy of Sciences, No. 19A Yuquan Road, Beijing, 100049 China

**Keywords:** Planetary science, Solid Earth sciences

## Abstract

Density imaging is a method that uses the inversion of the gravity and gravity gradient spectra in the wavenumber domain to create accurate 3D reconstructions of subsurface density distributions. This approach offers computational efficiency and rapid calculations. This research used preliminary inversions to examine the spectral characteristics of gravity and gravity gradient anomalies, as well as the resulting models, were scrutinized through preliminary inversions. 3D density imaging of gravity and gravity gradient was performed in the wavenumber domain using depth weighting on both noise-added and theoretical data, producing a density model that was consistent with the theoretical one. The technique was then used in the Decorah region of the United States, where 3D density imaging was performed and an examination of the properties of gravity and gravity gradient anomalies was conducted. The results showed where high-density Decorah complexes, low-density siliceous intrusive rocks, and high-density intrusive rock masses, were the distributed within the surrounding rock. Each of these provided comprehensive insights into the intrusive pathways to the rock mass. Thus, the appropriateness and effectiveness of the density imaging method were confirmed, supporting a deeper understanding of the structural division and geological evolution in the region.

## Introduction

For an extended period, scientific research has been enthralled by Earth’s profound subterranean density distribution, which presents a means of deciphering the dynamic evolution of our planet’s past and unleashing its untapped resource potential. Gravity and gravity gradient data are vital to this project since they are pivotal tools that reveal Earth’s density structure and hidden mineral resources. While low-frequency gravity anomaly data collection has historically been the main focus of conventional gravity prospecting, recent technical advancements have ushered in an era of unparalleled precision, that makes the meticulous measurement of gravity gradients possible. This innovation promises a new level of accuracy in sub-surface characterization by capturing high-frequency features as well as insights into the primary derivative of gravity anomalies. The fundamental function of gravity prospecting, which goes beyond its traditional role in mineral exploration, is at the centre of this study. It is an essential building block for analyzing Earth’s intricate structural composition, exploring the abundance of its mineral resources, and mapping the vast regions of local geology. Moreover, its applications span diverse realms, from archaeological exploration to hydrological assessments and engineering geological surveys. Gravity anomalies unveil the hidden distribution of Earth’s materials, shedding light on its geological tapestry. Simultaneously, gravity gradients provide improved resolution, and are useful for detecting sudden changes in the field source and revealing shallow anomalies. The synergy of multiple gradient datasets holds the potential to refine geological interpretations, unveiling new layers of insight^1,2^.

Comprehensive tensor gravity gradient measurement systems represent a revolutionary development in the current environment, emphasizing the vital significance of high-precision gravity and gravity gradient data inversion. Traditional gravity inversion is based on linear or non-linear methods, minimizes objective functions, and is frequently in line with least squares inversion theory principles. With the help of this persistent effort, significant research in gravity and gravity gradient inversion, has been conducted, enabling us to determine the parameters of geological models, dynamic interface depths, and the complex density distribution inside Earth’s geological framework^3–8^.

However, the journey of geophysical inversion is shadowed by non-uniqueness, requiring the careful application of constraints. To surmount this challenge, a judicious amalgamation of diverse a priori information becomes imperative. The inversion process gains momentum by utilizing physical property ranges, geological understanding, and related geophysical techniques. This combination of sophisticated inversion techniques and contextual constraints emphasizes how important it is to achieve realistic representations of Earth’s subterranean complexity. Some experts have improved the inversion technique based on elements such as adaptive pruning L-curve technique and hybrid PCG-bat algorithm, but they are mainly used for two-dimensional inversion. The research on three-dimensional physical property inversion is imminent^9–11^. In this context, density imaging in the wavenumber domain becomes a powerful tool that allows one to directly calculate the sub-surface density distribution from gravity anomalies. First of all, the wavenumber domain gravity orthogonal computation can significantly reduce storage space and increase computational speed, by converting complex pleated product computation in the spatial domain to a simple product computation in the wavenumber domain. This effectively increases computational efficiency. Previous studies have been conducted for many models, and wavenumber domain gravity normalization is a better-developed technical method^12–14^. While it is not dependent on a priori models, the wavenumber domain density imaging method exploits the advantages of wavenumber domain orthogonal computation. A wide range of approaches has been produced in the research history. For instance, the Cribb imaging technique uses the Fourier transform to reveal density distribution derived from vertical derivatives of observed gravity anomalies^15^, whereas the density equivalent distribution method proposed by Kobrunov and Varfolomeev, operates in the wavenumber domain and inverts density distribution grounded in gravity data spectra^16^. Fedi presented the DEXP approach, which uses extreme points to forecast the depth of anomalous structures^17^. A 3D correlation imaging technique was presented by Guo et al., that provides high spatial resolution for geological body occurrences and residual mass distribution^18^. Priezzhev’s method leverages rapid computation in the wavenumber domain, combining a priori information with gravity field data^19,20^. Kobrunov’s iterative inversion approach, based on functional representation, expeditiously produces sub-surface density and structural models^21^. Using depth scaling factors, Cui and Guo recently enhanced the wavenumber domain density imaging technique to produce high-resolution density models^22^. Previous studies on gravity and gravity gradient inversion have demonstrated the effectiveness of spatial domain inversion methods, however, they come with a cost in terms of computational time and space requirements. Meanwhile, frequency domain inversion methods struggle with limited depth resolution due to the rapid decay of gravity field spectra. Moreover, there is a clear research vacuum about the spectral characteristics of gravity gradient data.

Our study endeavours to address these voids by examining the spatial anomalies and spectral characteristics of gravity and gravity gradient data in the wavenumber domain of the theoretical model. Drawing on prior knowledge from gravity and gravity gradient spectra, we incorporate a depth weighting factor into the wavenumber domain density imaging method. This innovation aims to improve depth resolution, with the use of wavenumber domain gravity field inversion’s inherent computational efficiency. We also extend this novel methodology to gravity gradient data, taking advantage of its unmatched lateral resolution for density imaging. This study presents tangible outcomes rather than staying in the theoretical discourse domain. Our findings highlight the achievement of three-dimensional density models with high precision, that are obtained from the inversion of both gravity and gravity gradient data. We move beyond theory and apply our method to real-world data from the Decorah area in the United States, utilizing measured airborne gravity gradient data. The successful creation of a high-precision three-dimensional density model of subterranean space validates the effectiveness of our approach and increases its utility and applicability. In the pages that follow, we embark on a rigorous journey, delving into the opportunities and challenges that converge as we strive for high-density imaging precision. Through the utilization of high-precision gravity and gravity gradient data inversion, we aim to further explore the geological enigma of the Earth. Our method not only resolves existing questions but also provides the key to unlocking unprecedented depths of comprehension. As we navigate this scientific frontier, our vision encompasses revolutionizing resource management, reshaping exploration paradigms, and deepening our understanding of Earth’s hidden complexities.

## Methods

### Gravity and gradient forward modelling theory

**Figure 1:**
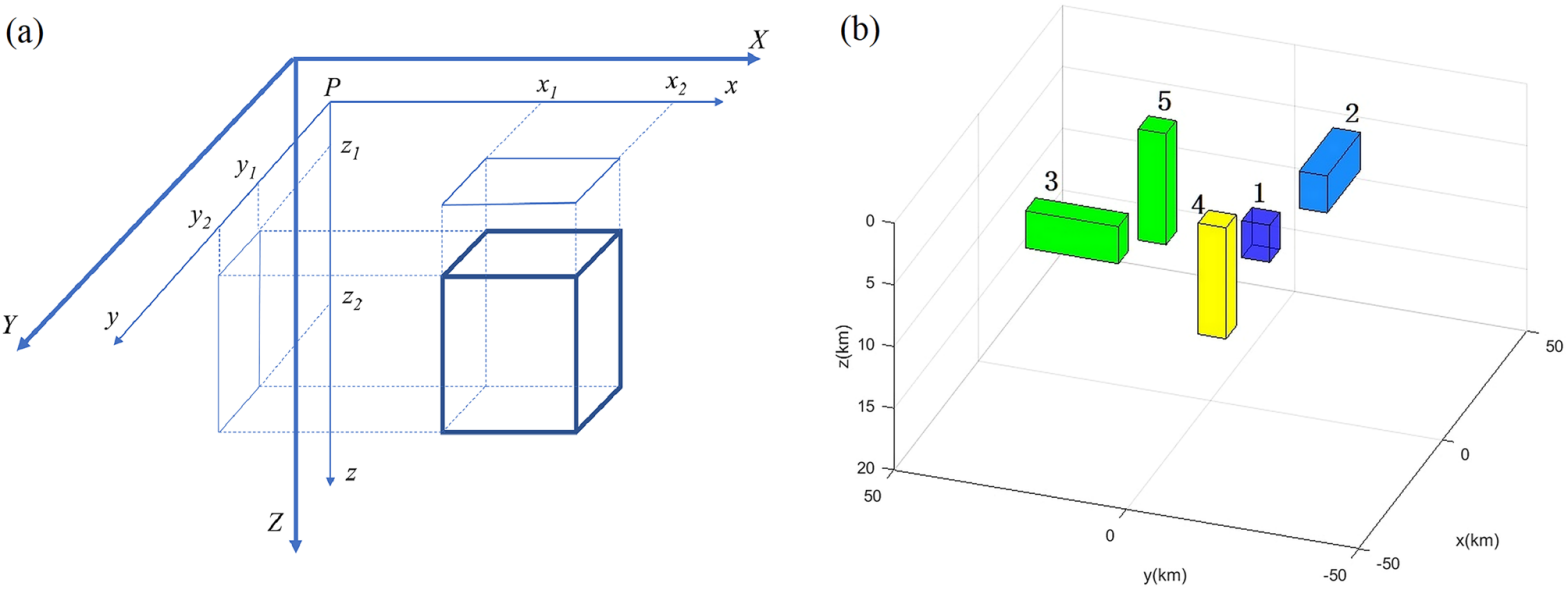
Rectangular prism model, (**a**) is Model I, and (**b**) is Model II.

The gravity anomaly formula utilizes the rectangular prism models as delineated by Blakely^23^ (Fig. 1a), and is confined to the limits of ${x}_{1}\le x\le {x}_{2}$, ${y}_{1}\le y\le {y}_{2}$ and ${z}_{1}\le z\le {z}_{2}$, as reported by Plouff^24^:

1 $$\begin{array}{c}\begin{array}{cc}{g}_{z}=& \gamma \rho \sum_{i=1}^{2} \sum_{j=1}^{2} \sum_{k=1}^{2} {\mu }_{ijk}\left[{z}_{k}{\text{arctan}}\frac{{x}_{i}{y}_{j}}{{z}_{k}{R}_{ijk}}-{x}_{i}{\text{log}}\left({R}_{ijk}+{y}_{j}\right)\right.\\ & \left.-{y}_{j}{\text{log}}\left({R}_{ijk}+{x}_{i}\right)\right]\end{array},\end{array}$$

where, ${g}_{z}$ is the gravity anomaly, $\gamma$ is 6.67 × 10^–11^ N·m^2^/kg^2^, which is the gravitation constant, $\rho$ is the density.

$$\begin{array}{l}{R}_{ijk}=\sqrt{{x}_{i}^{2}+{y}_{j}^{2}+{z}_{k}^{2}}\\ {\mu }_{ijk}=(-1{)}^{i}(-1{)}^{j}(-1{)}^{k}\end{array}.$$

The wavenumber domain spectrum of both gravity and gravity gradient is derived via Fast Fourier Transform (FFT), thereby yielding the gravity anomaly spectrum, as previously demonstrated^19^.

2 $$\overline{T} \begin{array}{*{20}c} { = \left[ {\begin{array}{*{20}c} {\frac{{\partial g_{x} }}{\partial x}} & {\frac{{\partial g_{x} }}{\partial y}} & {\frac{{\partial g_{x} }}{\partial z}} \\ {\frac{{\partial g_{y} }}{\partial x}} & {\frac{{\partial g_{y} }}{\partial y}} & {\frac{{\partial g_{y} }}{\partial z}} \\ {\frac{{\partial g_{z} }}{\partial x}} & {\frac{{\partial g_{z} }}{\partial y}} & {\frac{{\partial g_{z} }}{\partial z}} \\ \end{array} } \right] = \left[ {\begin{array}{*{20}l} {T_{xx} } \hfill & {T_{xy} } \hfill & {T_{xz} } \hfill \\ {T_{yx} } \hfill & {T_{yy} } \hfill & {T_{yz} } \hfill \\ {T_{zx} } \hfill & {T_{zy} } \hfill & {T_{zz} } \hfill \\ \end{array} } \right],} \\ \end{array}$$

where ${T}_{pq}\left(p,q=x,y,z\right)$ is each component of the gradient whose second-order derivatives satisfy Laplace’s equation, ${T}_{xx}+{T}_{yy}+{T}_{zz}=0$, and with ${T}_{xy}={T}_{yx}, {T}_{xz}={T}_{zx}, {T}_{yz}={T}_{zy}$ .Due to these two facts, only five components of the gradient are independent. Each gravity gradient component responds uniquely to the size, shape, and thickness of the density anomaly, providing a wide range of constraints in the interpretation process.

The gravity and its gradient are converted to the wavenumber domain to obtain its spectrum and the spectrum of the gravity anomaly ${g}_{z}$ is obtained according to the Fourier transform theory:

3 $$\begin{array}{c}G\left({k}_{x},{k}_{y}\right)=\underset{-\infty }{\overset{\infty }{\int }}\underset{-\infty }{\overset{\infty }{\int }}g_{z}\left(x,y\right){e}^{-i\left({k}_{x}x+{k}_{y}y\right)}dxdy,\end{array}$$

where $G\left({k}_{x},{k}_{y}\right)$ represents the spectrum of the gravity anomaly. ${k}_{x}$ and ${k}_{y}$ are the wavenumbers along the *x*-axis and *y*-axis, respectively. The gravity gradient formula in the wavenumber domain calculated is following^25^:

4 $$\begin{array}{c}{\Gamma }_{ij}\left({k}_{x},{k}_{y}\right)=\left[K({k}_{x},{k}_{y})G\left({k}_{x},{k}_{y}\right)]\right],\end{array}$$

where $$[K({k}_{x},{k}_{y})]=\left[\begin{array}{ccc}\frac{-{k}_{x}^{2}}{|k|}& \frac{-{k}_{x}{k}_{y}}{|k|}& -i{k}_{x}\\ \frac{-{k}_{x}{k}_{y}}{|k|}& \frac{-{k}_{y}^{2}}{|k|}& -i{k}_{y}\\ -i{k}_{x}& -i{k}_{y}& |k|\end{array}\right]$$.

### 3D density imaging theory of gravity and gravity gradient in the wavenumber domain

According to Priezzhev the density imaging formula in the wavenumber domain reveals the following^19,20,22^:

5 $$\begin{array}{*{20}c} {\rho (x,y,z) = F^{ - 1} \left[ {\frac{1}{2\pi \gamma }\frac{{(n + 1)^{n + 1} }}{n!}z^{n} e^{ - nkz} k^{n + 1} G\left( {k_{x} ,k_{y} ,0} \right)} \right].} \\ \end{array}$$

The constant $n$ ($1<n<10$) reflects the filter resolution, with larger values indicating higher resolution. $z$ is the inversion depth, ${\text{k}}$ is the wavenumber, and $\rho$ is the density of the mass body at a given point. Based on the conversion relationship between the gravity gradient anomaly spectrum and gravity anomaly spectrum^25^, the formula for inverting density distribution according to the gravity gradient anomaly spectrum can be derived through Formula (2), such as

6 $$\begin{array}{*{20}c} {\rho (x,y,z) = F^{ - 1} \left[ {\frac{1}{2\pi \gamma }\frac{{(n + 1)^{n + 1} }}{n!}z^{n} e^{ - nkz} k^{n + 1} \frac{{{\Gamma }_{ij} \left( {k_{x} ,k_{y} } \right)}}{{K\left( {k_{x} ,k_{y} } \right)}}} \right].} \\ \end{array}$$

### Forward modelling of gravity and gravity gradient of rectangular prism model

**Figure 2:**
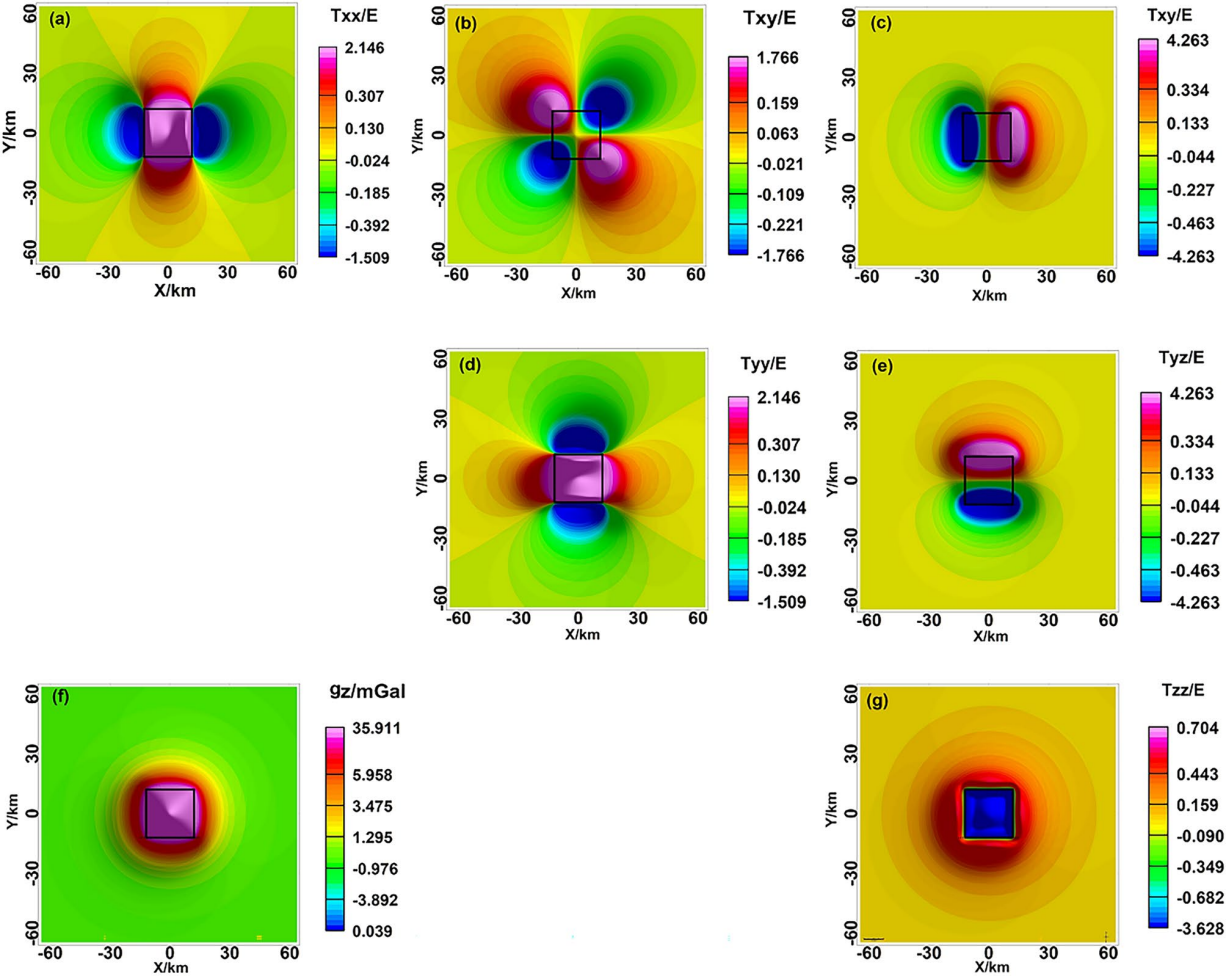
Gravity and gravity gradient anomalies of Model I, (**a–g**) the anomalies of ${T}_{xx}$, ${T}_{xy}$, ${T}_{xz}$, ${T}_{yy}$, ${T}_{yz}$, ${g}_{z}$, ${T}_{zz}$, the black line is the model.

We simulated Model I to verify the relationship between gravity, gravity gradient, and the model. The anomalies in gravity and gravity gradient were linked to the edge, boundary, angle, and mass centre of the anomalous mass^26^ (Fig. 2). The boundaries were delineated by the zero value of ${T}_{xx}$ and ${T}_{yy}$, which corresponded to the east–west and north–south variations of gravity, respectively. The corner point was determined by the extreme value of ${T}_{xy}$. The anomalous axis in the north–south and east–west directions were represented by ${T}_{xz}$ and ${T}_{yz}$, respectively, with their extreme values indicating the boundaries. The abnormal centre was indicated by the extreme value of ${g}_{z}$ and ${T}_{zz}$ with, ${T}_{zz}$ showing higher resolution than $\Delta g$ and its zero-value corresponding to the boundary^27^. The gravity anomaly spectrum displayed rapid attenuation and fluctuation, while the gravity gradient spectrum displayed slower attenuation, pronounced periodicity, and a discernible response in the ${k}_{x}$ and ${k}_{y}$ directions. The spectrum characteristics of the gravity gradient were closely linked to the orientation of each component, with ${T}_{xx}$ and ${T}_{xy}$ displaying rapid attenuation and unsuitability for further inversion.

### Analogous spectral features of gravity anomaly and gradient amplitude spectrum and determination of buried depth

**Figure 3:**
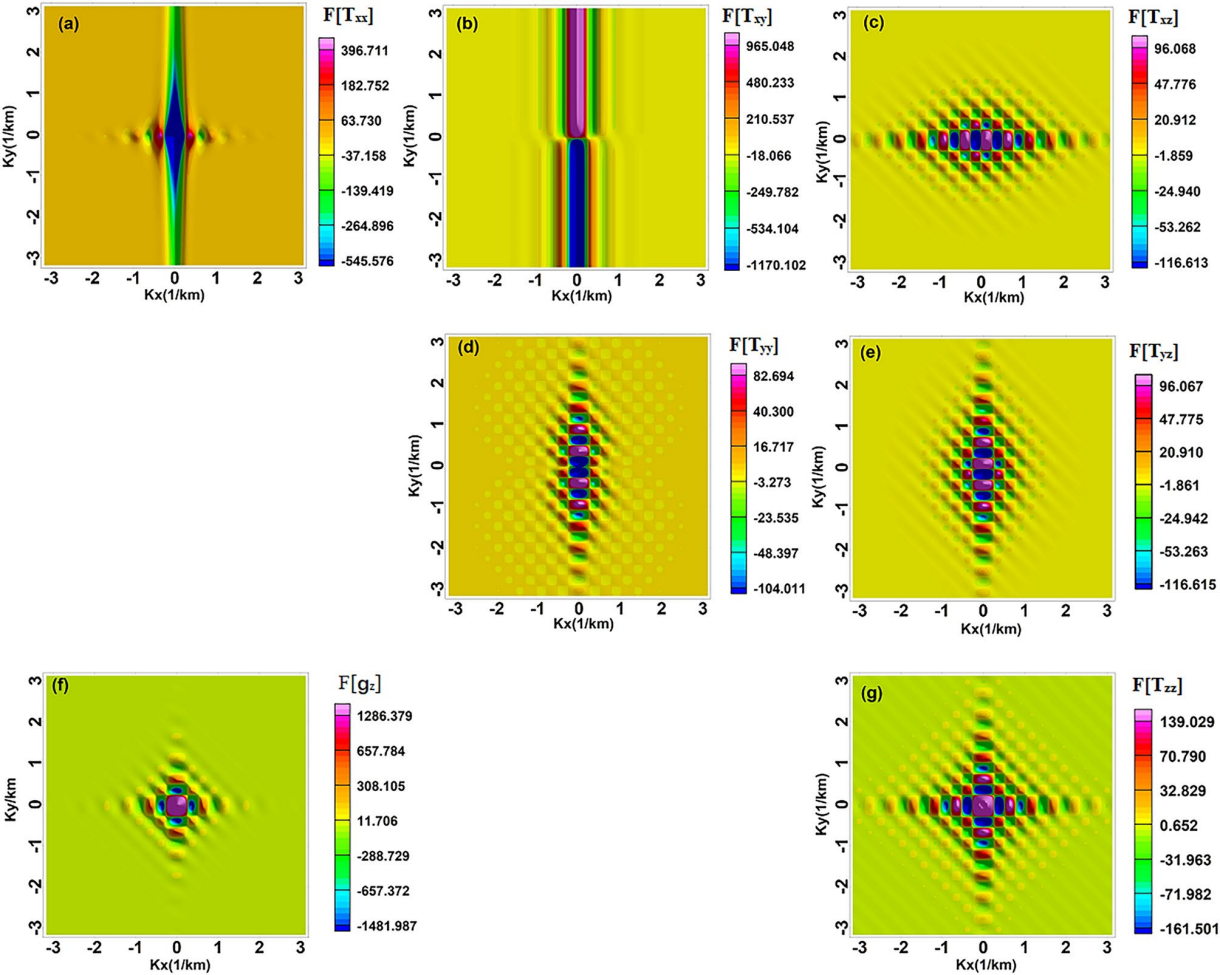
The amplitude spectrum of the gravity gradient, (**a–g**) the anomalies of $F[ {T}_{xx}]$, $F[ {T}_{xy}]$, $F[{T}_{xz}]$, ${F[T}_{yy}]$, $F[{T}_{yz}]$, $F[{g}_{z}]$, ${F[T}_{zz}]$

**Figure 4:**
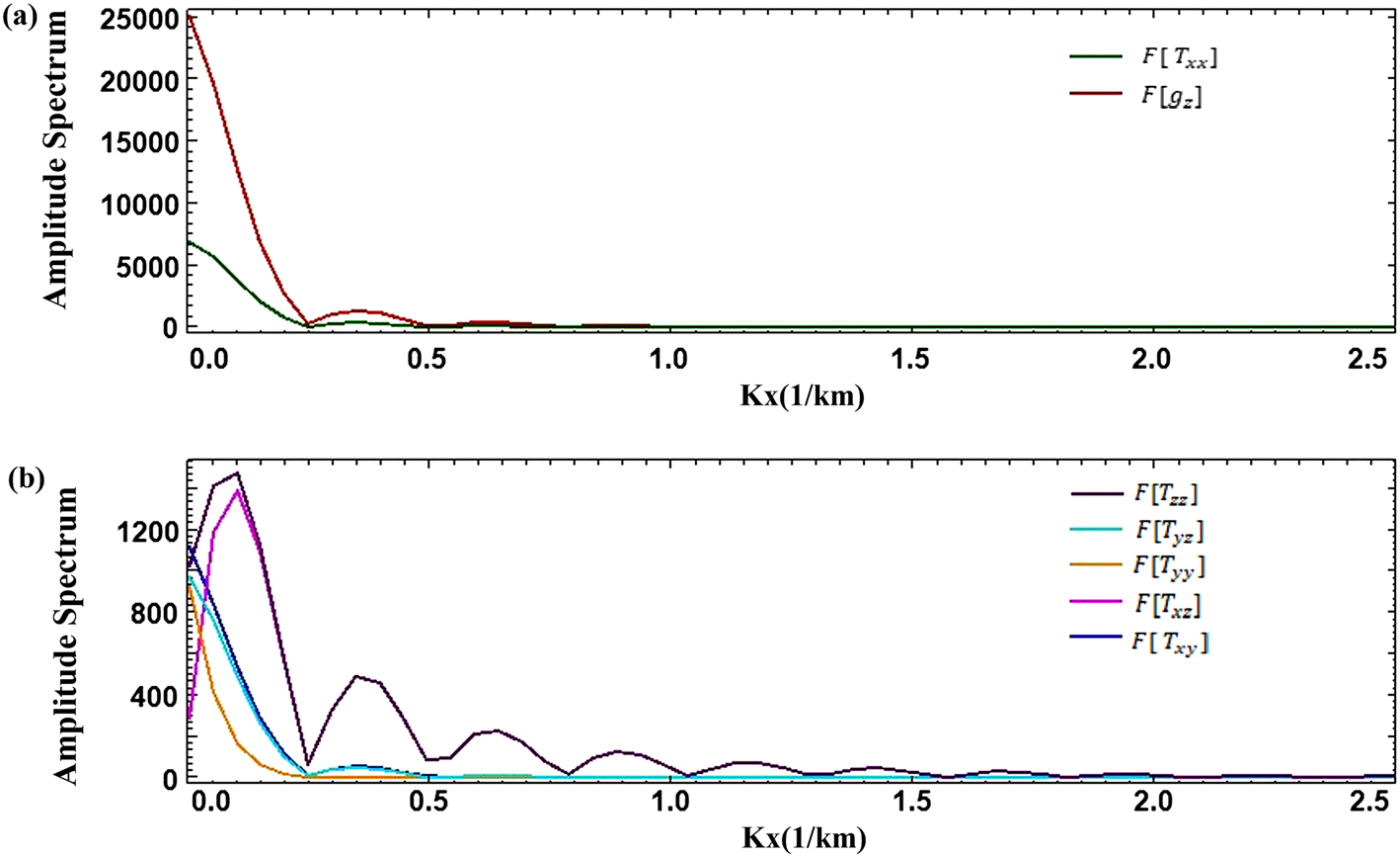
Amplitude spectrum profile of gravity anomaly and gravity gradient (${k}_{x}$ = 0).

The distributional attributes of the anomaly amplitude are revealed by the amplitude spectrum, which captures the frequency-dependent fluctuations in the gravity anomaly amplitude spectrum. The gravity anomaly and gradient amplitude spectrum exhibit analogous spectral features, which decay rapidly, and the wavenumber corresponding to the zero value varies periodically^28^ (Fig. 3). The fluctuation-type amplitude spectrum curve is mainly related to the horizontal width of the geologic body, based on the characteristics of the amplitude spectrum curve that provide a preliminary judgment of the morphology of the anomalous body. The spacing of wavenumbers between the amplitude’s zero-value points is closely related to the horizontal width of the body. The rectangular prism model can be inverted and the half-width in the horizontal direction can be derived from the wave value at the first minimum of the spectrum.

7 $$\begin{array}{c}B=\frac{\pi }{\Delta k},\end{array}$$

where B is the width of the body, $\pi$ is the constant, which is 3.14, and $\mathrm{\Delta k}$ is the spacing of wavenumbers between the zero-value points of the amplitude. The gravity anomaly spectra of 3D and 2D bodies are equivalent^29^, allowing for the extraction of profiles at ${k}_{y}$ = 0 for both the gravity anomaly and gradient amplitude spectra. Due to the small gradient amplitude spectrum, two profiles were required. The first minimum point of the amplitude spectrum corresponds to a wave value of 0.247 (Fig. 4), resulting in a calculated half-width of 12.7 km, which closely matches the actual width of 12 km.

The buried depth of a model can be determined by calculating the slope of its average radial logarithmic power spectrum, obtained by logarithmically transforming the average radial power spectrum^30^.

8 $$\begin{array}{c}{h}_{t}=-\frac{{\text{ln}}E\left({r}_{2}\right)-{\text{ln}}E\left({r}_{1}\right)}{2\left({r}_{2}-{r}_{1}\right)},\end{array}$$

where ${h}_{t}$ is depth, $E(r)$ is the average radial power spectrum, and r is the radial frequency.

### Noise tolerance evaluation of density imaging method using gravity gradient data

**Figure 5:**
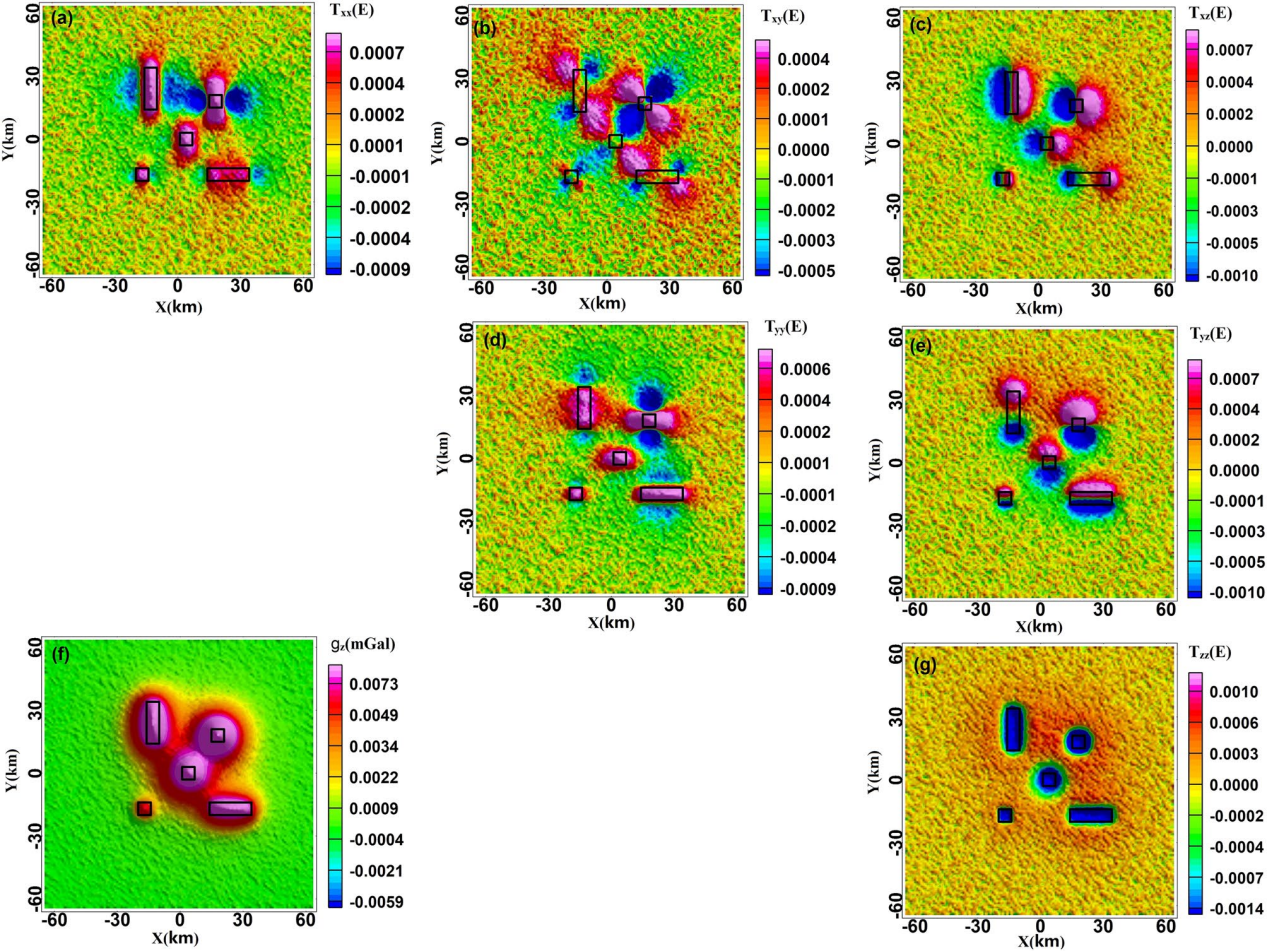
Gravity and gravity gradient anomalies of Model II (**a–g**) the anomalies of ${T}_{xx}$, ${T}_{xy}$, ${T}_{xz}$, ${T}_{yy}$, ${T}_{yz}$, ${g}_{z}$, ${T}_{zz}$, the black line is the model.

To test the density imaging method’s noise tolerance, we added 10% random noise and calculated the theoretical forward gravity and gravity gradient anomalies of Model II (Fig. 1b). It is commonly known that the gravity anomaly was unable to accurately represent the model boundary; however, the gravity gradient anomalies aligned well with the inflexion point or model boundary, with ${T}_{zz}$ providing a clear outline of the model position. The presence of noise produced fuzzy characteristic lines for the gravity and gravity gradient data but had no discernible effect on the fundamental properties of the anomaly that corresponded to the model body (Fig. 5). Furthermore, we performed a comparative analysis of the computational speed of Model I and Model II. The spatial domain model orthogonalization is the analytical computation of the rectangular model and the wavenumber domain computation is done using Matlab 2016b compiled software. The computer parameters are as follows: 32 GB of the Random Access Memory (RAM) and a 12th Gen Intel(R) Core (TM) i7-12700 K 3.60 GHz the Central Processing Unit (CPU). The comparison results show that, particularly for large and complex models, the orthorectified modelling in the wavenumber domain is faster than modelling in the spatial domain, improving the inversion efficiency (Table 1).

**Table 1:** Comparison of forward duration of gravity and gravity gradient in space domain and wavenumber domain.

	Forward duration in the space domain	Forward duration in the wavenumber domain	Forward modelling speed advantage in the wavenumber domain
Model I	0.08 s	0.03 s	Increase by 62.5%
Model II	0.26 s	0.05 s	Increase by 80.7%

The more complex the model is, the more obvious the velocity advantage in the wavenumber domain is.

### Iterative inversion based on depth weighting

The proposed method’s effectiveness and noise immunity were verified by inverting forward modelling and noise-added data. The model’s depth and width were initially evaluated using the gravity spectrum as a density imaging constraint. To enhance longitudinal resolution, we introduced Commer’s depth weighting function^31^, W(z) is a spatial gradient weighting function that improves inversion results by introducing a priori depth information. This function has been widely used since it was proposed^32–34^.

9 $$\begin{array}{c}W\left(z\right)=\frac{\alpha +{\text{exp}}\left[\frac{{d}_{1}}{dz}\left(z-{z}_{c1}\right)\right]}{1+{\text{exp}}\left[\frac{{d}_{1}}{dz}\left(z-{z}_{c1}\right)\right]}-\frac{\alpha +{\text{exp}}\left[\frac{{d}_{2}}{dz}\left(z-{z}_{c2}\right)\right]}{1+{\text{exp}}\left[\frac{{d}_{2}}{dz}\left(z-{z}_{c2}\right)\right]},\end{array}$$

where z is the centre depth, $dz$ is the inverse domain, $\alpha$ is the empirical value that determines the weights at the near-surface and is generally taken as $\alpha =0.001$ to overcome the skin effect. ${z}_{c1}, {z}_{c2}$ are the depth of the top and bottom of the model. ${d}_{1}$ and ${d}_{2}$ are the interface constraint factors for the upper and lower interfaces, respectively. The iterative inversion formula with the depth weighting function shows the following:

10 $$\begin{array}{c}{\rho }_{i+1}={\rho }_{i}+W\left(z\right)\times \Delta {\rho }_{i},\end{array}$$

where, ${\rho }_{i}$ is the density at i iteration, ${\rho }_{i+1}$ is at i + 1 iteration, $\Delta {\rho }_{i}$ is the correction. The subterranean domain to be inverted is partitioned into *N* horizontal layers, each layer is divided into $m\times n$ orthogonal prisms.

**Figure 6:**
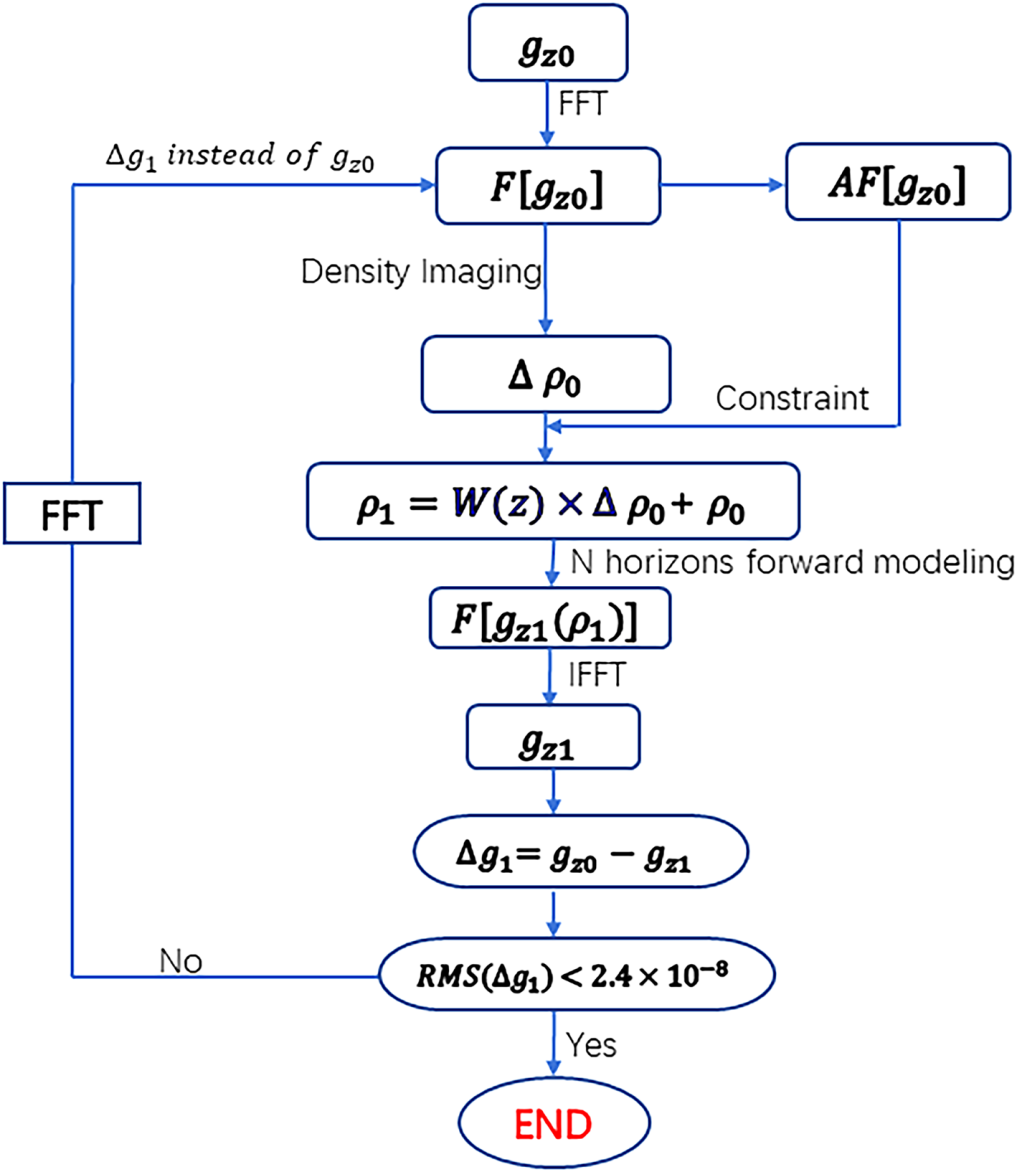
Iterative density imaging with amplitude spectrum inversion constraints for gravity anomaly modelling.

As illustrated in Fig. 6, the specific iterative process entails the following steps:

- *Step 1* Begin by creating an initial density file, denoted as ${\rho }_{0}$, with an initial value of zero. The dimensions of this file are configured to correspond with the range of gravity anomalies.
- *Step 2* Next, calculate the spectrum of observed gravity anomalies (or gravity gradients), and use Eq. (8) to infer depth-related data from the obtained spectrum.
- *Step 3* Apply Eq. (5) (or Eq. 6) to density imaging calculations using the gravity anomaly (or gravity gradient anomaly) spectrum, ultimately yielding $\Delta {\rho }_{0}$.
- *Step 4* Combining the results of Step 2 with other geological information, initiate the first iteration of the density distribution, denoted as ${\rho }_{1}$. This is accomplished by taking the depths obtained in the second step and combining them with other geologic information, combining the depth constraint function of Eq. (9), and then applying Eq. (10).
- *Step 5* Model the density distribution ${\rho }_{1}$ in the entire spatial domain orthogonal to the wavenumber domain. This facilitates the generation of its gravity anomaly spectrum (or gravity gradient anomaly spectrum), followed by a Fourier inverse transformation to obtain the computed gravity anomalies (or gravity gradient anomalies).
- *Step 6* After obtaining the computed values, compare them to the observed values. Calculate the root mean square (RMS) difference and evaluate the fitting results. If the difference in gravity anomaly fitting is less than 2.4 × 10^−8^ (or gravity gradient fitting difference is less than 1 × 10^−3^), terminate the iteration. Otherwise, consider the fitting difference as the observed gravity anomaly (or gravity gradient anomaly) and repeat steps two to six in the iteration process.

In this study, the model inversion depth is 20 km, the subsurface is divided into 40 rectangular layers, the grid of each layer is 128 × 128, and the inversion time is 15 s. Compared with traditional inversion methods such as least squares^35,36^, there is a very significant improvement in computational speed while both achieve computational accuracy. The top burial depth of the model is obtained as 0.2 km through the spectrum information, and the bottom burial depth is estimated as 15 km through the different parameters, and the addition of the depth weighting factor can control the depth of the model inversion to avoid the epidermal effect and the depth is too large. Iterating over each prism within the strata is accomplished as follows. This information-independent approach enables direct initialization of the density to zero. The specific iterative process is shown in Fig. 6. We present a novel density imaging methodology utilizing a flowchart approach to iteratively refine density models for gravity anomaly modelling. Notably, amplitude spectrum inversion results serve as key constraints driving the iterative process. We increment density models with each imaging outcome until the residual error between the model gravity forward value and the observed gravity anomaly satisfies predetermined accuracy thresholds, the RMS of $\Delta {g}_{1}$ is 2.4 × 10^–8^, while it is 1 × 10^−3^ of ${T}_{zz}$ in the presented inverse problems, halting the iteration. This approach offers an efficient and effective means to refine density models for accurate gravity anomaly modelling.

### Comparative analysis of density imaging results for Model I and Model II using gravity and gravity gradient data

**Figure 7:**
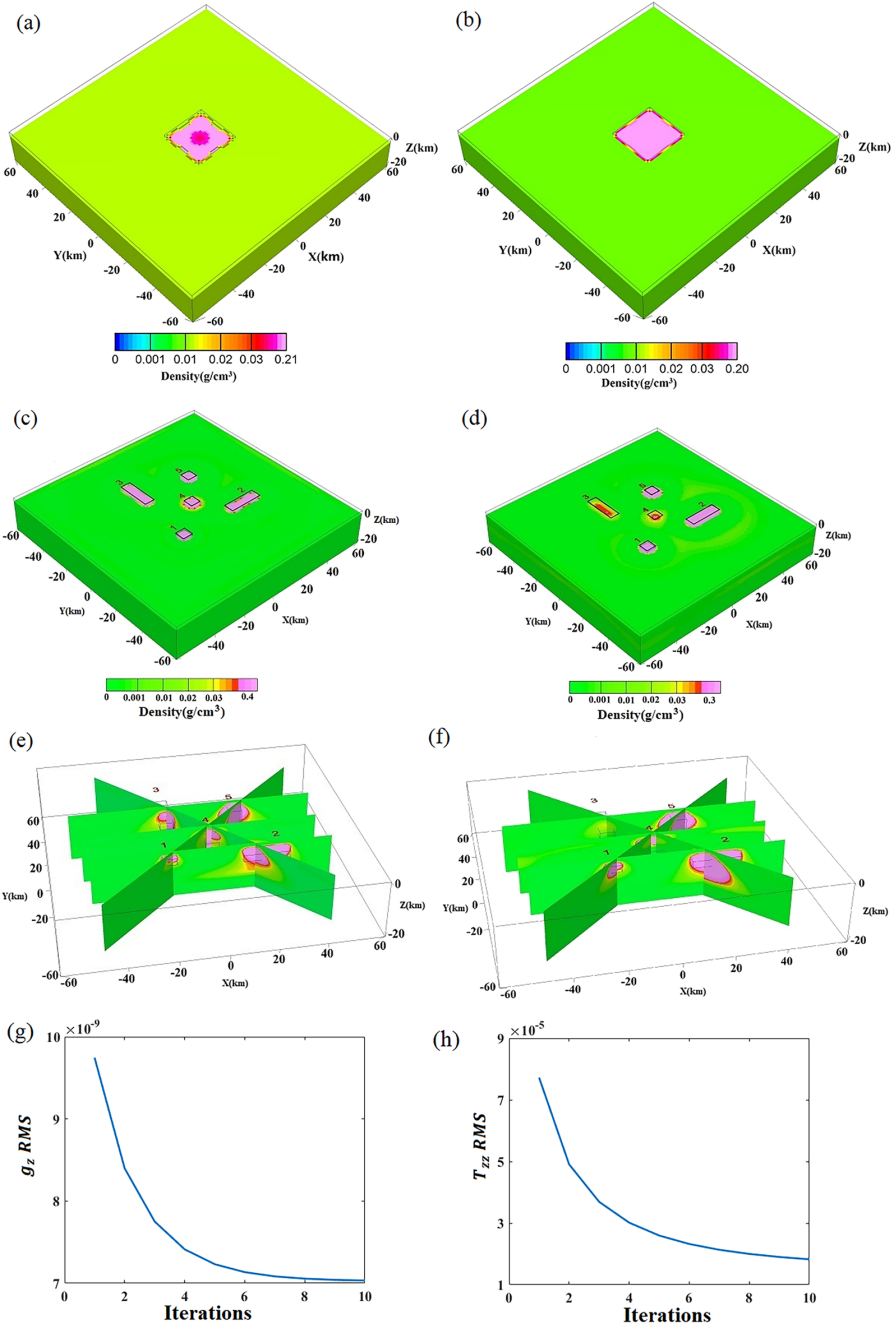
${g}_{z}$/${T}_{zz}$ 3D density imaging results of Model I and II. (**a,b**) are inversion results of Model I, ${g}_{z}$ and ${T}_{zz}$ results, respectively; (**c–f**) are results of Model II, (**c,e**) is the 3D body and slices of ${g}_{z}$ result, respectively. (**d,f**) are about ${T}_{zz}$ result. (**g,h**) the RMS of ${g}_{z}$ and ${T}_{zz}$ iterations.

The wavenumber domain computation of density imaging for Model I and Model II as tabulated in Table 2 is presented here. The resultant 3D density imaging outcomes based on ${g}_{z}$ and ${T}_{zz}$ are depicted in Fig. 7. The inversion effects of the data from both models are impeccable, and their corresponding theoretical positions have been accurately determined. The horizontal positions of the inversion results of the data sets are delineated in Fig. 7, where the actual positions of the models are represented by black boxes, and the boundaries of the models are clearly illustrated. The vertical profile of the inversion result is evident that the position of the anomaly body in both data sets is in excellent agreement with the positions of the model. We note that the higher density in Model 4 leads to a slight tailing phenomenon in Model 5.

**Table 2:** Model parameters.

Model No	Model type	Parameter/km	Parameter/km	Density/kg/m^3^
Model I	Single rectangular prism	(− 12, − 12, 1)	(12, 12, 10)	150
Model II	Composite rectangular prism	(− 20, − 20, 1)	(− 14, − 14, 4)	100
		(14, − 20, 3)	(34, − 14, 6)	300
		(− 16, 14, 4)	(− 10, 34, 7)	500
		(1, − 3, 6)	(7, 3, 15)	700
		(15, 15, 2)	(21, 21, 11)	800

### Analysis of the accuracy and noise resistance of 3D density imaging using gravity gradient data

The 3D density imaging results of gravity gradients, apart from ${g}_{z}$ and ${T}_{zz}$ exhibit a higher rate of false anomalies (Table 3, Fig. 7). This phenomenon is attributed to the derivative of the data, which leads to distortions in ${T}_{xx}$ and ${T}_{xz}$, ${T}_{yy}$ and ${T}_{yz}$, and ${T}_{xy}$ in the *x* and *y* direction. In contrast, the 3D density imaging outcomes of ${g}_{z}$ and ${T}_{zz}$ display a remarkable level of accuracy. Table 3 presents the residuals obtained from the comparison between modelled values of ${g}_{z}$ and ${T}_{zz}$, both orthogonal to the theoretical values derived from density imaging through ${g}_{z}$ and ${T}_{zz}$. The table demonstrates the exceptional accuracy of ${g}_{z}$ and ${T}_{zz}$ in both Model I and Model II. In Model I, the RMS of ${g}_{z}$ residual is 0.004, while the RMS for ${T}_{zz}$ is a mere 2.3 × 10^–4^. Similarly, in Model II, these values further improve to 7.03 × 10^–9^ and 1.83 × 10^–5^ respectively. When introducing a 10% random noise to the inversion results^37^, a slight enhancement is observed, yielding an RMS of ${g}_{z}$ residual of 7.09 × 10^–9^ and 9.75 × 10^–5^ for the two models. Despite this noise, the residuals comfortably adhere to the computational accuracy prerequisites, which mandate ${g}_{z}$ residual within 1 × 10^–4^ and ${T}_{zz}$ residual within 1 × 10^–5^. We also tested the inversion results introducing a 20% and 50% random noise for Model II and the RMS of ${g}_{z}$ and ${T}_{zz}$ residuals were 7.29 × 10^–9^, 1.92 × 10^–4^ of the former data, 8.57 × 10^–9^, and 4.84 × 10^–4^ of the later, respectively. It follows that the results of 3D density imaging, even with the added noise, remain in accord with those of the original model, with the dense values predominantly situated within the model area. The assessment of the density imaging reveals a near-consistent forward modelling value of the inverted density model and the residual error of the theoretical value, indicative of the robust noise resistance properties of the method, and, ${T}_{zz}$ is even better at it. Verification of the method’s efficacy was performed in the Decorah region of the United States.

**Table 3:** Evaluation of the effect of gravity/gravity gradient 3D density imaging.

Parameters involved in density imaging		Maximum residual value	Minimum residual value	Residual average	RMS
Model I	${g}_{z}$	− 0.002	− 0.026	− 0.013	0.004
	${T}_{zz}$	0.001	− 0.002	− 5 × 10^–4^	2.3 × 10^–4^
Model II	${g}_{z}$	1.795 × 10^–3^	1.793 × 10^–3^	1.79 × 10^–3^	7.03 × 10^–9^
	${T}_{zz}$	4.3 × 10^–4^	− 1.45 × 10^–7^	− 3.03 × 10^–5^	1.83 × 10^–5^
	${g}_{z}$ with 10% noise	2 × 10^–3^	1.58 × 10^–3^	1.8 × 10^–3^	7.09 × 10^–9^
	${T}_{zz}$ with 10% noise	6.42 × 10^–4^	− 0.015	− 2.89 × 10^–5^	9.75 × 10^–5^

The residual between the forward gravity anomaly of the theoretical model proposed in this article and the result obtained from model inversion in the wavenumber domain serves as a crucial evaluation metric.

## Results and conclusions

### Full tensor gravity and gravity gradient measurements for 3D density imaging in the Decorah region

The Decorah region is rich in mineral resources and offers a promising target for exploration due to its vast sedimentary terrain. Bell Geospace has conducted meticulous gravity and gravity gradient measurements, capturing comprehensive full tensor data, by utilizing cutting-edge technologies. Three-dimensional density imaging has been generated to delineate sub-surface density distributions by using a wavenumber domain method. The efficacy of this approach has been validated through rigorous analyses.

### Gravity and gravity gradient anomalies in the Decorah

**Figure 8:**
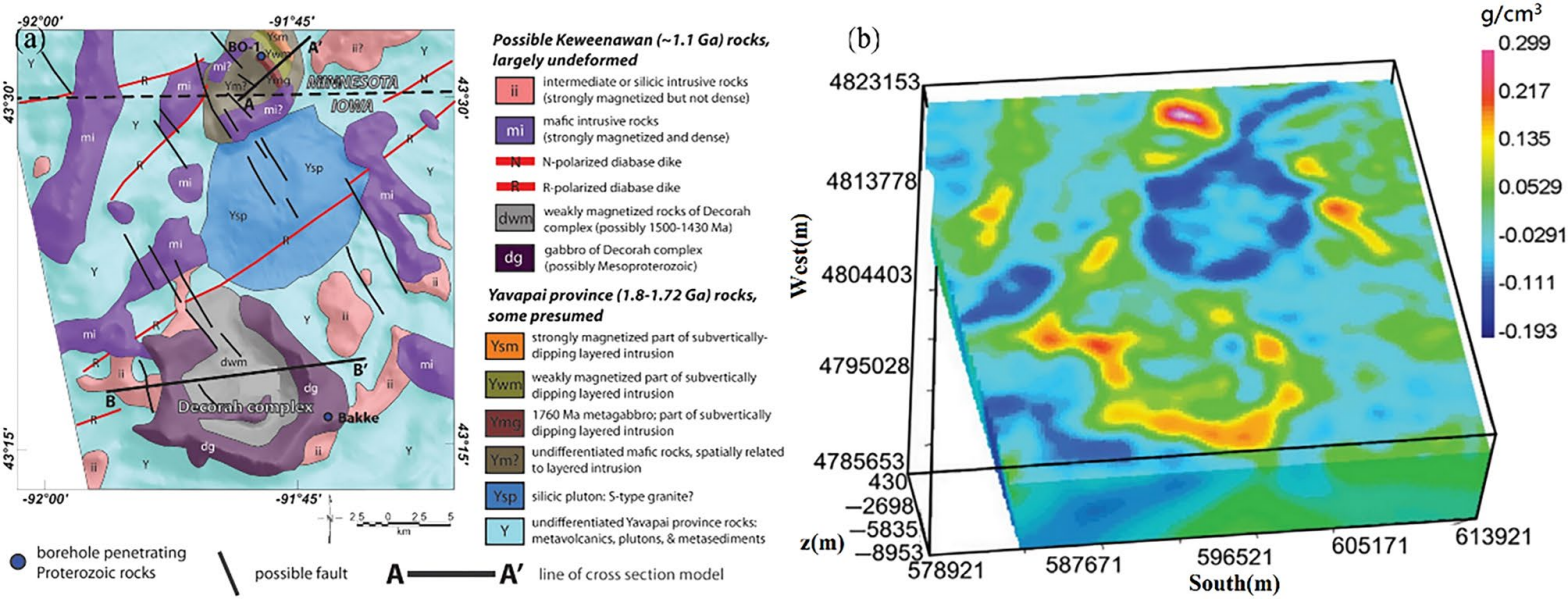
The geological and density model of the Decorah, (**a**) is a Geologic map^38^, and (**b**) is a 3D density model^39^**.**

Anomalies related to both gravity and gravity gradient are caused by variations in density within the Earth’s interior. The latter is sensitive to variations in shallow density differences, whereas the former represents information from deep field sources. Comparison of the two allows for a comprehensive understanding of the density distribution within a specific area. Gaussian regional filtering, with a 2 km wavelength cutoff is used to identify the target anomalies and eliminate the impact of sedimentary caprock. The area under investigation, referred to as the Northeastern Iowa intrusive complex, is composed of a lower-density siliceous intrusion in the middle and the comparatively dense Decorah complex in the south (Fig. 8a). Within this area, several small, relatively dense mafic and siliceous rocks have intruded into the surrounding low-density rock^38,39^.

### Analysis of gravity anomalies in the Decorah region

**Figure 9:**
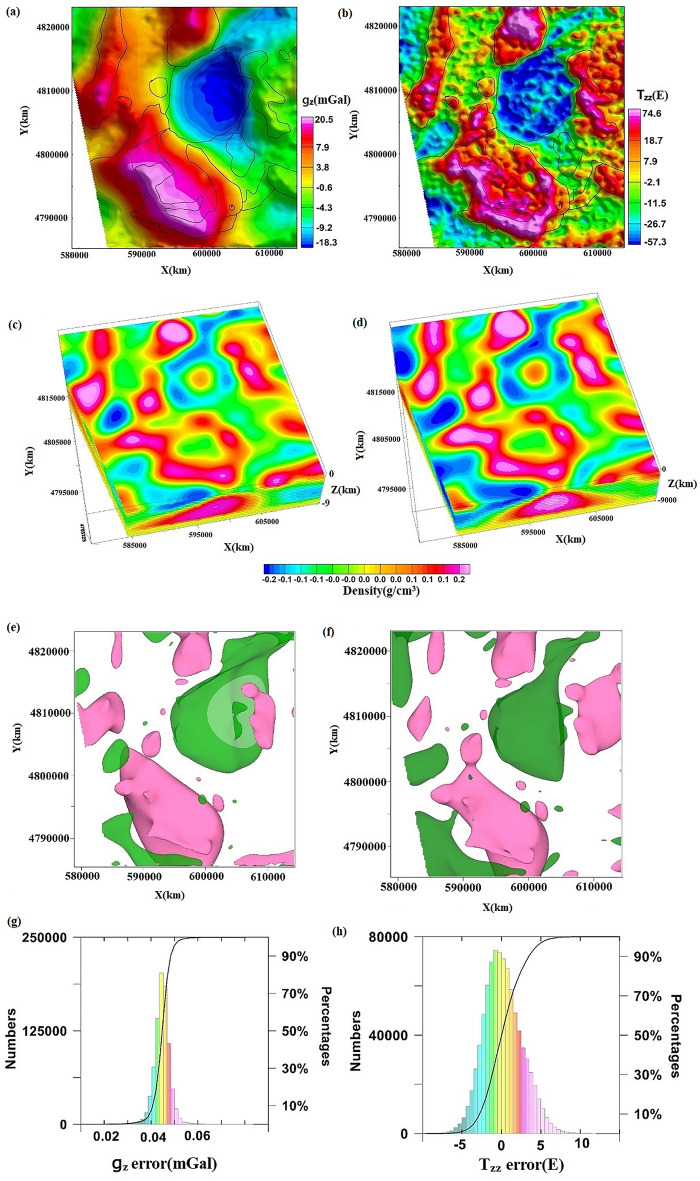
Measured gravity and gravity gradient anomalies in the Decorah. Blacklines are tectonic lines. (**a**) ${g}_{z}$, (**b**) ${T}_{zz}$, (**c,e**) are the inversion results of ${g}_{z}$, (**c**) density voxel, (**e**) isosurfaces, pink marks ρ is 0.11, green marks ρ is  − 0.1. (**d,f**) are the inversion results of ${T}_{zz}$, (**d**) density voxel, (**f**) isosurfaces, pink marks ρ is 0.2, green marks ρ is − 0.18.(**g,h**) represent the residuals between the forward and observed values of the model obtained from ${g}_{z}$ and ${T}_{zz}$ inversion, respectively.

Between December 2012 and January 2013, Bell GeoSpace used an aerial gravity full tensor gradient measurement system to survey the subject area. A total of 94 EW survey lines, spaced at 400 m intervals, and 9 connecting lines, spaced at 4000 m intervals, were measured. The resulting gravity and gravity gradient anomalies are displayed in Fig. 9a,b, respectively. Examination of the ${g}_{z}$ values revealed a broad range of low gravity anomalies in the northeast region, corresponding to siliceous intrusion, as confirmed by geological mapping. Numerous high-value anomaly traps and belts were found in the western portion of the study area. These belts and traps extend north-to-east, with a limited high-value anomaly range in the northern sector, and an expansive, high-amplitude, high-value range from southwest to south, which is indicative of the Decorah Complex. Further analysis of the ${T}_{zz}$ map exposed several local anomalies, with the eastern region displaying a prominent high-value area and the southern portion exhibiting a distinct outline of the high-value area. Additional anomalies with minor amplitudes were found in flat regions. Cumulatively, analysis of the gravity and gravity gradient data facilitated the division of the study area into multiple structural units. Density imaging was conducted leveraging ${g}_{z}$ and ${T}_{zz}$, with the sub-surface inversion at the depth of 0–8 km, layer interval of 150 m, 20 iterations, and sub-surface model grid points of (930 × 997 × 53).

### Physical property analysis of the Decorah region

The 3D density body horizon at a depth of 1250 m in the study region was determined using the Tikhonov regularization inversion method^38^ (Fig. 8b). The horizon at the same depth was calculated using a novel technique presented in this study (Fig. 9c,d), with mostly consistent findings. An analysis of the Decorah’s geologic map revealed the location of different intrusive rock bodies. On the east and north faces, two anomaly bodies with a relatively small range and high density were identified as mafic intrusion bodies; on the east and north sides, a large-scale SW-trending Decorah complex was found (Fig. 9e,f). The variation in sub-surface 3D density structure was clearly illustrated, revealing the intrusion process of the bodies from bottom to top. Together with the detected entities, the density model’s iso-surfaces correspond to distinct geological structural units with minor anomalies. The inversion results were reliable, with residuals conforming to a normal distribution and having a standard deviation of 0.003 mGal for ${g}_{z}$ and 3.4 E for ${T}_{zz}$ (Fig. 9g,h). A superior high-precision density model of the research region was generated by utilizing a novel 3D density imaging technique in the wavenumber domain. This allowed for a comprehensive understanding of the geological distribution and mineral resources within this area.

## Conclusion

In this study, we conducted an in-depth analysis of gravity and gravity gradient anomalies in the spatial and wavenumber domains. Leveraging the efficient computational capabilities in the wavenumber domain, we obtained a priori information on the model through amplitude spectrum inversion and introduced depth weighting to constrain the wavenumber-domain 3D density imaging method. This enabled us to quickly and effectively create an accurate 3D underground density model, which we then validated using measurements taken in real-world environments. Through a rigorous examination of the model results and spectrum characteristics, we conclude that ${g}_{z}$ and ${T}_{zz}$ demonstrate superior 3D density imaging inversion with a significant noise immunity and depth imaging effect. Additionally, the 3D density imaging inversion utilizing ${g}_{z}$ and ${T}_{zz}$ in the Decorah area of the United States unveiled the distribution of intrusive rocks and their intrusion path.

This work highlights the effectiveness of the 3D density inversion method in the wavenumber domain and provides crucial insights for further research. Our future work will be focused on maximizing the superiority of gravity and gravity gradient through joint inversion and improving the accuracy of the sub-surface 3D density model.

## Data Availability

The model datasets used and/or analyzed during the current study are available from the corresponding author upon reasonable request. The survey data that support the findings of this study are available from Bell Geospace but restrictions apply to the availability of these data, which were used under license for the current study, and therefore not publicly available. Data can, however, be obtained from the authors upon reasonable request and with permission from Bell Geospace.
